# Osteopontin Expression Is Associated with the Poor Prognosis in Patients with Locally Advanced Esophageal Squamous Cell Carcinoma Receiving Preoperative Chemoradiotherapy

**DOI:** 10.1155/2018/9098215

**Published:** 2018-04-30

**Authors:** Tai-Jan Chiu, Hung-I Lu, Chang-Han Chen, Wan-Ting Huang, Yu-Ming Wang, Wei-Che Lin, Shau-Hsuan Li

**Affiliations:** ^1^Department of Hematology-Oncology, Kaohsiung Chang Gung Memorial Hospital and Chang Gung University College of Medicine, Kaohsiung, Taiwan; ^2^Department of Thoracic & Cardiovascular Surgery, Kaohsiung Chang Gung Memorial Hospital and Chang Gung University College of Medicine, Kaohsiung, Taiwan; ^3^Institute for Translational Research in Biomedicine, Kaohsiung Chang Gung Memorial Hospital, Kaohsiung, Taiwan; ^4^Department of Applied Chemistry and Graduate Institute of Biomedicine and Biomedical Technology, National Chi Nan University, Taiwan; ^5^Center for Infectious Disease and Cancer Research, Kaohsiung Medical University, Kaohsiung, Taiwan; ^6^Department of Pathology, Kaohsiung Chang Gung Memorial Hospital and Chang Gung University College of Medicine, Kaohsiung, Taiwan; ^7^Department of Radiation Oncology, Kaohsiung Chang Gung Memorial Hospital and Chang Gung University College of Medicine, Kaohsiung, Taiwan; ^8^Department of Diagnostic Radiology, Kaohsiung Chang Gung Memorial Hospital and Chang Gung University College of Medicine, Kaohsiung, Taiwan

## Abstract

**Background:**

The osteopontin has been involved in therapeutic resistance in a variety of cancers. But, the significance of osteopontin expression on the prognosis of patients with locally advanced esophageal squamous cell carcinoma (ESCC) receiving chemoradiotherapy is unclear.

**Methods:**

In 80 patients with locally advanced ESCC receiving preoperative chemoradiotherapy between 1999 and 2012, osteopontin expression was evaluated by immunohistochemistry and correlated with treatment outcome. The functional role of osteopontin in ESCC cell lines was determined by osteopontin-mediated siRNA.

**Results:**

Osteopontin expression and clinical T4 classification were significantly associated with poor pathological complete response. Univariate analyses demonstrated that osteopontin overexpression and clinical T classification, T4, were significantly associated with worse overall survival and disease-free survival. In multivariate comparison, osteopontin overexpression and clinical T classification, T4, represented the independent adverse prognosticator. In ESCC cell lines, endogenous osteopontin depletion by osteopontin-mediated siRNA increased sensitivity to cisplatin. Osteopontin expression is independently correlated with the response of chemoradiotherapy and prognosis of patients with locally advanced ESCC receiving preoperative chemoradiotherapy.

**Conclusions:**

Our results suggest that osteopontin may be a potential therapeutic target for patients with ESCC treated with preoperative chemoradiotherapy.

## 1. Background

The prognosis of patients with locally advanced esophageal squamous cell carcinoma (ESCC) receiving surgery alone is poor [1, 2]. To improve treatment outcome, a multimodality treatment using combined chemoradiotherapy followed by esophagectomy has been suggested for these patients [3]. However, the necessity of the esophagectomy after chemoradiotherapy remains largely undefined. Previous phase III clinical trials [3, 4] revealed that esophagectomy may be unnecessary for those patients who respond well to chemoradiotherapy. After preoperative chemoradiotherapy, 20–40% patients can achieve pathological complete response (pCR) and thus have significantly improved survival [5–7]. However, there is still a large portion of patients who cannot respond well to chemoradiotherapy [6]. Therefore, it is important to explore the signaling pathway involved in the resistance of chemoradiotherapy and recognize patients who are likely to respond to chemoradiotherapy to spare them the potential perioperative complications.

Osteopontin is an arginine-glycine-aspartate-containing adhesive glycoprotein whose expression is elevated in various types of cancer including ESCC [8, 9]. Importantly, osteopontin has recently been reported to be related to the resistance of anticancer therapy in breast cancer [10], colon cancer [11], hepatocellular carcinoma [12], and oral cancer [13]. However, the significance of osteopontin expression on the prognosis of patients with locally advanced ESCC receiving chemoradiotherapy remains unclear.

Thus, we evaluated the osteopontin expression by immunohistochemistry and investigated its role in 80 patients with locally advanced ESCC treated with preoperative chemoradiotherapy.

## 2. Methods

### 2.1. Patient Population

We retrospectively reviewed patients with ESCC who received preoperative chemoradiotherapy followed by esophagectomy at Kaohsiung Chang Gung Memorial Hospital between January 1999 and December 2012. This study was approved by the Institutional Review Board of Chang Gung Memorial Hospital. All patients selected for the present study were required to have available pretreatment specimens of biopsy for immunohistochemistry. During this period, 80 patients were identified. Computed tomography (CT) scan of the chest and abdomen or/and endoscopic ultrasound (EUS) were performed for staging. Patients were evaluated by a multidisciplinary team including a thoracic surgeon, a radiologist, a radiation oncologist, a medical oncologist, and a gastroenterologist. The clinical staging was determined according to the 7th American Joint Committee on Cancer (AJCC) staging system. Overall survival (OS) was calculated from the date of diagnosis until death or last follow-up. Disease-free survival (DFS) was computed from the time of surgery to the recurrence or death from any cause without evidence of recurrence.

### 2.2. Treatment Plan

The protocol of preoperative chemoradiotherapy was described as previously [2, 6]. Within 3-4 weeks following the end of irradiation, CT scan was performed to assess the treatment response. The multidisciplinary team reviewed the clinical information to determine if the lesions were resectable. If the lesions were classified as resectable, surgery was advised approximately 6–10 weeks after the end of preoperative chemoradiotherapy. Patients undergoing surgery had a radical esophagectomy with cervical esophagogastrostomy or Ivor Lewis esophagectomy with intrathoracic anastomosis, two-field lymphadenectomy, reconstruction of the digestive tract with gastric tube, and pylorus drainage procedures. pCR was defined as the complete disappearance of all viable cancer cells in all surgical specimens including the primary esophageal tumor and lymph nodes.

### 2.3. Immunohistochemistry (IHC)

IHC staining was performed using an immunoperoxidase technique. Staining was performed on slides (4 *μ*m) of formalin-fixed, paraffin-embedded tissue sections with primary antibodies against osteopontin (AKm2A1, 1 : 100). Briefly, after deparaffinization and rehydration, slides were subjected to heat-induced epitope retrieval in 10 mM citrate buffer (pH 6.0) in a hot water bath (95°C) for 20 minutes. Endogenous peroxidase activity was blocked for 15 minutes in 0.3% hydrogen peroxide. After blocking with 1% goat serum for one hour at room temperature, the sections were incubated with primary antibodies for one hour at room temperature.

Immunodetection was performed using the LSAB2 kit (Dako, Carpinteria, CA) followed by 3-3′-diaminobenzidine for color development and hematoxylin for counterstaining. The staining assessment was independently carried out by two pathologists (S.L.W. and W.T.H.) without any information about clinicopathological features or prognosis. To investigate the expression of osteopontin, ten fields within the tumor were selected, and expression in 1000 tumor cells (100 cells per field) was evaluated using high-power (200x) microscopy. The osteopontin expression level was scored by using the 3-tier system: low expression, ≦10%; median expression, 11–50%; and high expression, >50% tumor cells with detectable immunoreaction in perinuclear and other cytoplasmic regions. When scores were classified into two groups for statistical analysis, “median expression” and “high expression” were combined as “overexpression” [9, 13].

### 2.4. Cell Culture and Transfection

Human ESCC cell lines TE10 and TE14 were obtained from European Collection of Cell Cultures (ECACC) and cultured in RPMI 1640 medium with 10% FBS, 1% (v/v) penicillin-streptomycin solution and maintained at 37°C in 5% CO2 humidified air. TE10 and TE14 cells (5 × 10^4^ cells) were seeded into 6-well dishes and cultured at 37°C in 5% CO2 humidified air. After 24 hours, si-Osteopontin and si-Control plasmids were transfected into the cells with lipofectamine 2000 reagent according to manufacturer's instructions, followed by further incubation for 24 hours at 37°C in 5% CO2. Then, cells were harvested for following western blotting.

### 2.5. Western Blotting Assay

Cell pellets were lysed in RIPA lysis buffers (1 mM Na3VO4, 25 mM NaF, and 1 × protease inhibitor cocktail protease inhibitor cocktail). Protein concentrations were determined by spectrophotometry. Sample was electrophoresed by 10% SDS-PAGE gel and followed by transferred to PVDF membranes. These membranes were then blocked with 5% nonfat dry milk for 1 h at room temperature and incubated with primary antibodies. Monoclonal antiantibodies, osteopontin and *β*-actin, were purchased from Santa Cruz and incubated with membrane at room temperature for 1 hour. HRP-conjugated secondary antibody was incubated at room temperature for 1 hour. The membrane was then developed using an enhanced chemiluminescence system and exposed to X-ray film.

### 2.6. Cell Viability Assay

Cells were plated onto 6 wells at 1 × 10^5^ cells/well and transfected with si-Control or si-Osteopontin plasmids and for following incubation overnight. Next day, transfectant cells were harvested and seeded onto 96 wells at 5 × 103 cells/well for overnight. Then cells were treated with or without 10 *μ*M of Cisplatin for 48 hours, and the cells' viability was determined by using MTT assay. All growth experiments were carried out in triplicate.

### 2.7. Drug Treatment

Cells were treated for the indicated time with cisplatin and osteopontin at the indicated concentration for assay of cell survival.

### 2.8. Statistical Analysis

For patient data, statistical analysis was performed using the SPSS 17 software package. The chi-square test or Fisher's exact test was used to compare data between the two groups. Multivariate analysis of pathologic complete response was performed by logistic regression. For survival analysis, the Kaplan–Meier method was used for univariate analysis, and the difference between survival curves was tested by a log-rank test. In a stepwise forward fashion, parameters were entered into Cox regression model to analyze their relative prognostic importance. For all analyses, two-sided tests of significance were used with *P* < 0.05 considered significant. For ESCC cell viability assay, statistical analyses were performed by one-way analysis of variance (ANOVA) with Tukey's adjustment for pairwise comparisons, using Prism (version 4.0) from Graph Pad. Data were mean  ±  SD from three independent trial and the *P* value less than 0.05 was considered significant

## 3. Results

### 3.1. Patient Characteristics

A total of 80 patients were collected in the study with a median age of 53 years (range, 37–77 years). Among them, 77 were men and 3 were women. The T classifications were T2 in 7 (9%) patients, T3 in 36 (45%) patients, and T4 in 37 (46%) patients. The N classifications were N0 in 20 (25%) patients, N1 in 25 (31%) patients, N2 in 25 (31%) patients, and N3 in 10 (13%) patients. Additional analyses according to AJCC 7th staging system demonstrated stage II tumor for 21 (26%) patients and stage III for 59 (74%) patients. Further analyses of histological grades showed a grade 1 lesion in 16 (20%) patients, grade 2 in 43 (54%) patients, and grade 3 in 21(26%) patients, respectively. Primary tumor location was found upper in 15 (19%) patients, middle in 31 (39%), and lower in 34 (42%). Osteopontin expression showed low expression in 42 (52%) patients, median expression in 15 (19%) patients, and high expression in 23 (29%) patients (Table 1).

At the time of analysis, the median periods of follow-up were 104 months (range, 62.6–143.9 months) for the 17 survivors and 21.5 months (range, 3.8–143.9 months) for all 80 patients. The 5-year overall and disease-free survival rates of these 80 patients were 28% and 23%, respectively. Among these 80 patients, 21 (26%) patients achieved pCR after preoperative chemoradiotherapy. The 5-year overall and disease-free survival rates were 67% and 65% in patients with pCR and 14% and 10% in patients without pCR.

### 3.2. Correlation between Clinicopathological Parameters and the Expression of Osteopontin

**Figure 1:**
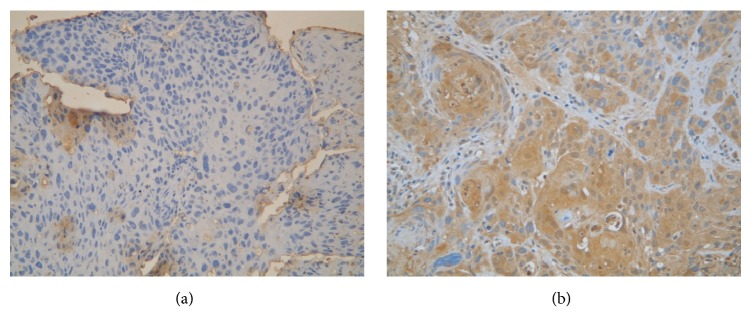
Immunohistochemical staining of osteopontin. (a) Representative example of low osteopontin expression in esophageal squamous cell carcinoma. (b) Representative example of osteopontin overexpression in esophageal squamous cell carcinoma.

Among the 80 patients collected, 42 patients (52%) showed “low expression” for osteopontin expression and the other “overexpression” (Figure 1). Osteopontin expression was not associated with any clinicopathologic parameters including age, primary tumor location, histological grade, AJCC 7th staging, T classification, and N classification (Table 2).

### 3.3. Correlation between Clinicopathological Parameters and Pathological Complete Response

The relationship between clinicopathological parameters and the response of chemoradiotherapy was summarized in Table 3. Osteopontin expression (*P* = 0.043) and T classification (*P* = 0.016) were significantly associated with pCR. The logistic model showed that osteopontin expression (low expression versus overexpression; *P* = 0.025, hazard ratio: 3.687, 95% confidence interval: 1.176–11.564) and T classification (T2/3 versus T4; *P* = 0.011, hazard ratio: 4.602, 95% confidence interval: 1.410–15.019) were independently correlated with pCR after chemoradiotherapy.

### 3.4. Survival Analyses

**Figure 2:**
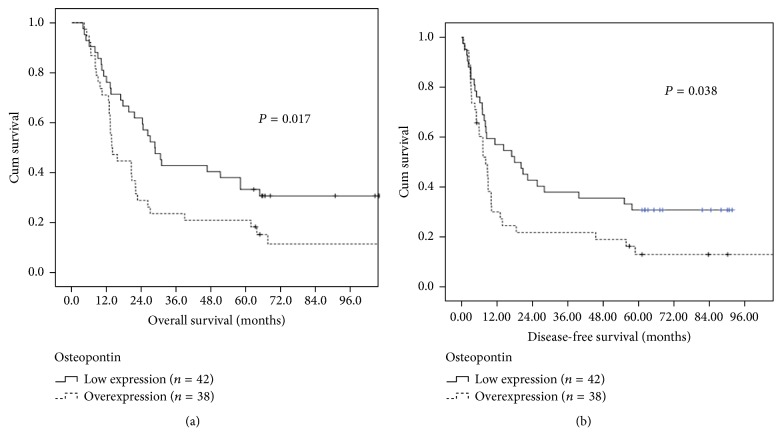
(a) Overall survival according to osteopontin expression. (b) Disease-free survival according to osteopontin expression.

Correlations of clinicopathological parameters and osteopontin expression with overall survival (OS) and disease-free survival (DFS) were summarized in Table 4. Univariate analyses showed that osteopontin overexpression (*P* = 0.017; Figure 2(a)) and clinical T classification, T4 (*P* = 0.024), were significantly associated with worse overall survival. Additionally, osteopontin overexpression (*P* = 0.038; Figure 2(b)) and clinical T classification, T4 (*P* = 0.035), were also significantly associated with inferior disease-free survival. In multivariate comparison, osteopontin overexpression (*P* = 0.004, hazard ratio: 2.171, 95% confidence interval: 1.287–3.661) and clinical T classification, T4 (*P* = 0.005, hazard ratio: 2.121, 95% confidence interval: 1.259–3.575), remained independently associated with worse overall survival. For disease-free survival, osteopontin overexpression (*P* = 0.022, hazard ratio: 1.828, 95% confidence interval: 1.093–3.060) and clinical T classification, T4 (*P* = 0.02, hazard ratio: 1.833, 95% confidence interval: 1.099–3.057), represented an independent adverse prognosticator. The 5-year overall and disease-free survival rates were 21% and 13% in patients with osteopontin overexpression and 33% and 31% in patients with low osteopontin expression, respectively.

### 3.5. Endogenous Osteopontin Depletion by siRNA Sensitizes Cytotoxicity to Cisplatin in Esophageal Squamous Cell Carcinoma Cells

**Figure 3:**
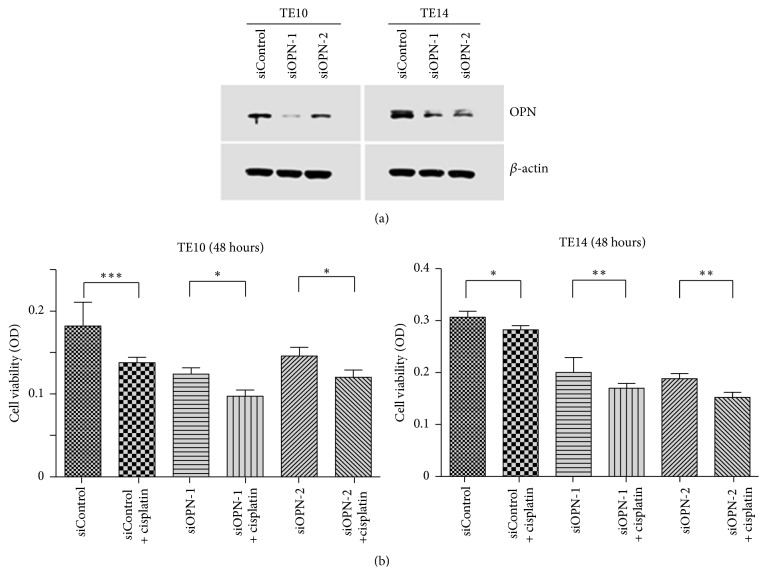
Endogenous osteopontin depletion by osteopontin siRNA sensitized the cytotoxicity to cisplatin in esophageal squamous cell carcinoma cell lines. (a) The endogenous expression level of osteopontin was determined by western blotting in TE10 and TE14 cells transfected with si-Control or si-Osteopontin. (b) TE10 and TE14 cells transfected with si-Control or si-Osteopontin were incubated with 10 *μ*M cisplatin for 48 hours, and their viability was measured and compared to that of untreated respective cells. OPN: osteopontin. ^*∗*^*P* < 0.05; ^*∗∗*^*P* < 0.01; ^*∗∗∗*^*P* < 0.001.

The expression level of osteopontin was determined by western blotting in TE10 and TE14 cell transiently transfected with si-Control or si-Osteopontin (Figure 3(a)). TE10 and TE14 cells transfected with si-Control or si-Osteopontin cells were incubated with or without 10 *μ*M of cisplatin for 48 hours. The viability of cells transfected with si-Osteopontin was significantly decreased compared to cells transfected with si-Control (Figure 3(b)). Together, these results demonstrate that osteopontin expression influences the response of ESCC cell lines to cisplatin treatment.

### 3.6. Osteopontin Promoted Esophageal Squamous Cell Carcinoma Cell Proliferation and Cisplatin Resistance

**Figure 4:**
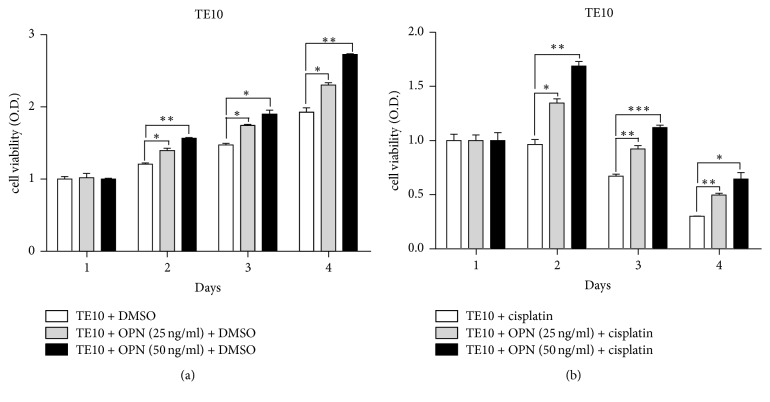
Osteopontin promoted cell proliferation and drove cisplatin resistance in an ESCC cell line. (a) TE10 cells stimulated with OPN protein promoted cell growth. TE10 cells were treated with indicated concentrations of OPN, and cell growth was analyzed on days 1–4 by MTT assay. Data were normalized against the OD570 value on day 1 of each treatment. The results represent the mean ± SD of three independent experiments. (b) OPN affected the chemosensitivity of ESCC cells to cisplatin. TE10 cells were cultured in the cisplatin and/or OPN in a dose-dependent manner, and their viability was measured. ^*∗*^*P* < 0.05; ^*∗∗*^*P* < 0.01; ^*∗∗∗*^*P* < 0.001.

To understand the role of osteopontin in ESCC cell proliferation, recombinant human osteopontin was executed to TE10 cells to determine if increased osteopontin protein could promote proliferation in TE10 cells. The proliferation rate was significantly increased in matricellular-osteopontin in a dose-dependent manner in SAS cells (Figure 4(a)). This result demonstrates that one of the major roles of osteopontin is to promote growth of ESCC cells.

To get further insight into the biological effect that osteopontin might enhance chemoresistance to cisplatin, we performed MTT assay to assess cell viability in cells incubated with osteopontin and cisplatin treatment. As shown in Figure 4(b), TE10 cells with osteopontin were found to be significantly more resistance to cisplatin than the cells obtained from cisplatin control group.

## 4. Discussion

This study confirmed immunohistochemical osteopontin overexpression predicted poor prognosis in locally advanced ESCC patients treated with preoperative chemoradiotherapy. In our study, osteopontin was overexpression in 48 percent of locally advanced esophageal squamous cell carcinoma patients and this finding was consistent with Kita's study in which osteopontin was immunohistochemical positive expression in 48 percent of esophageal squamous cell carcinoma patients after esophagectomy with lymph node dissection [9]. However, in the present study, osteopontin overexpression was not associated with any clinicopathological factors. Osteopontin expression was significantly associated with lymph node metastasis and lymphatic invasion in Kita and Shimada studies [9, 14]. Wu et al. also showed that the more severe the clinical stage, the higher the frequency of overexpression of osteopontin mRNA [8]. Zhang et al. found that expression of OPN-c is significantly elevated in ESCCs and OPN-c was significantly associated with pathological T stage (*P* = 0.038) and overall stage (*P* = 0.023) [15]. In their study, they investigated the relationship between osteopontin expression and clinicopathological factors in stage I to stage IV ESCC patients after operation by immunohistochemical staining and enzyme immunoassay. None of these patients underwent preoperative chemotherapy and/or radiotherapy. In our study, all patients were AJCC stage II/III ESCC and thus received preoperative chemoradiotherapy followed by esophagectomy and lymph node dissection. Comparing to previous studies, our patient population was relatively limited in late AJCC tumor stage and advanced T and N stage tumor patients' factors.

For stage II or III localized thoracic ESCC, the recent meta-analysis showed that preoperative concurrent chemoradiotherapy followed by surgery reduces locoregional recurrence and has significant survival benefit compared to surgery alone [16]. The achievement of a pathological complete response is commonly considered an important prognostic factor after preoperative chemoradiation [17]. Until now, there is no clear prognostic factor to predict treatment response of preoperative chemoradiation. The overexpression of osteopontin in tumor tissues has been associated with a worse prognosis in a variety of malignancies, including squamous cell carcinoma of the esophagus [14, 18] and head and neck [13], but only few studies have addressed the role of osteopontin in chemoradiotherapy resistance [19, 20], especially in ESCC. In this study, IHC staining showed that osteopontin overexpression correlated with a more resistance to cisplatin-based chemoradiotherapy and has a significantly worse OS than those whose tumors had low expressed osteopontin. These clinical results indicate that osteopontin may be involved in the cisplatin resistance in ESCC. For further confirmation of correlation of osteopontin and cisplatin resistance, we also tried to knock down osteopontin in ESCC cell lines, TE10 and TE14, to study the involvement of osteopontin in cisplatin resistance. We observed that lower osteopontin expression significantly reversed cisplatin resistance in ESCC cell lines. These results suggested that osteopontin overexpression enhanced cisplatin resistance in ESCC.

The osteopontin is an integrin-binding protein and involved in a variety of physiological cellular functions, including the process of tumorigenesis and metastases. Molecular mechanisms that define the role of osteopontin in chemotherapy resistance have not been completely elucidated, although several mechanisms have been studied. One recent study in small cell lung cancer also demonstrated that osteopontin increased chemoresistance to cisplatin in SBC-3 cells by suppressing bcl-2 protein downregulation [19]. In our previous oral squamous cell carcinoma study, we also found that overexpression of osteopontin predicts a poor response and survival to cisplatin-based induction chemotherapy followed by CCRT in patients with locally advanced oral squamous cell carcinoma [13], and cisplatin-based induction chemotherapy is one of the standard treatment modalities for patients with locally advanced head and neck squamous cell carcinoma [21]. In the advanced head and neck squamous cell carcinoma, Aurora-A is upregulated by osteopontin stimulation [22]. In ESCC cells, overexpression of Aurora-A inhibits the cisplatin- or UV irradiation-induced apoptosis [23]. Therefore, it is speculated that one of possibilities might be that activation of osteopontin-Aurora-A signaling confers attenuation of cisplatin and radiation induced apoptosis in ESCC.

Our study has important limitations. First, the present study was a retrospective analysis. Second, our observations were limited by the relatively small number of patients.

In conclusion, we found that the overexpression of osteopontin predicts a poor response and survival in patients with locally advanced ESCC receiving preoperative chemoradiotherapy. Furthermore, inhibition of osteopontin can sensitize esophageal cancer cells to cisplatin. Therefore, osteopontin may be a promising target for patients with ESCC who receive multimodality treatment.

## Figures and Tables

**Table 1:** Clinicopathologic features of 80 patients with locally advanced esophageal squamous cell carcinoma receiving preoperative chemoradiotherapy.

Parameters	Number of cases (percentage)
Age (years) (mean: 54.3, median: 53, range 37–77)	
<50	25 (31%)
50≦Age < 60	28 (35%)
60≦Age < 70	22 (28%)
70≦Age	5 (6%)
Sex	
Male	77 (96%)
Female	3 (4%)
Clinical 7th AJCC stage	
II	21 (26%)
III	59 (74%)
Clinical T classification	
T2	7 (9%)
T3	36 (45%)
T4	37 (46%)
Clinical N classification	
N0	20 (25%)
N1	25 (31%)
N2	25 (31%)
N3	10 (13%)
Tumor grade	
Grade 1	16 (20%)
Grade 2	43 (54%)
Grade 3	21 (26%)
Primary tumor location	
Upper	15 (19%)
Middle	31 (39%)
Lower	34 (42%)
Osteopontin expression	
Low	42 (52%)
Median	15 (19%)
High	23 (29%)
pCR	
Absent	59 (74%)
Present	21 (26%)

pCR, pathological complete response.

**Table 2:** Associations between osteopontin expression and clinicopathologic parameters.

Parameters	Osteopontin expression		
	Low expression	Overexpression	*P* value
Age			
<53 y/o	22	17	0.50
≧53 y/o	20	21	
Clinical 7th AJCC stage			
II	9	12	0.30
III	33	26	
Clinical T classification			
T2/3	21	22	0.48
T4	21	16	
Clinical N classification			
N0	9	11	0.44
N1/2/3	33	27	
Clinical N classification			
N0/1	27	18	0.13
N2/3	15	20	
Tumor grade			
Grade 1/2	30	29	0.62
Grade 3	12	9	
Primary tumor location			
Upper/middle	24	22	0.95
Lower	18	16	

**Table 3:** Associations between pathological complete response and clinicopathologic parameters.

Parameters	Pathological complete response		
	Present	Absent	*P* value
Age			
<53 y/o	9	30	0.53
≧53 y/o	12	29	
Clinical 7th AJCC stage			
II	8	13	0.15
III	13	46	
Clinical T classification			
T2/3	16	27	0.016^*∗*^
T4	5	32	
Clinical N classification			
N0	6	14	0.66
N1/2/3	15	45	
Clinical N classification			
N0/1	15	30	0.10
N2/3	6	29	
Tumor grade			
Grade 1/2	15	44	0.78
Grade 3	6	15	
Primary tumor location			
Upper/middle	9	37	0.11
Lower	12	22	
Osteopontin			
Low expression	15	27	0.043^*∗*^
Overexpression	6	32	

^*∗*^Statistically significant.

**Table 4:** Results of univariate log-rank analysis of prognostic factors for overall survival and disease-free survival in 80 patients with locally advanced esophageal squamous cell carcinoma receiving preoperative chemoradiotherapy.

Factors	Number of patients	Overall survival (OS)		Disease-free survival (DFS)	
		5-year OS rate (%)	*P* value	5-year DFS rate (%)	*P* value
Age					
<53 y/o	39	26%	0.73	21%	0.56
≧53 y/o	41	29%		25%	
Osteopontin					
Low expression	42	33%	0.017^*∗*^	31%	0.038^*∗*^
Overexpression	38	21%		13%	
Clinical 7th AJCC stage					
II	21	43%	0.13	29%	0.27
III	59	22%		21%	
Clinical T classification					
T2/3	43	35%	0.024^*∗*^	28%	0.035^*∗*^
T4	37	19%		16%	
Clinical N classification					
N0	20	35%	0.46	25%	0.70
N1/2/3	60	25%		22%	
Clinical N classification					
N0/1	45	33%	0.064	25%	0.15
N2/3	35	20%		20%	
Tumor grade					
Grade 1/2	59	25%	0.40	19%	0.38
Grade 3	21	33%		33%	
Primary tumor location					
Upper/middle	46	28%	0.61	23%	0.60
Lower	34	27%		23%	

pCR, pathological complete response. ^*∗*^Statistically significant.
